# Ochsner Obstetrics and Gynecology Simulation Program: A Review of the Literature and Description of a Multidisciplinary Simulation Program Targeting Management of Obstetric Emergencies

**DOI:** 10.31486/toj.20.0014

**Published:** 2020

**Authors:** Wyeth Lawson, Michael Smith, Brigid McCue

**Affiliations:** ^1^ Department of Obstetrics and Gynecology, Ochsner Clinic Foundation, New Orleans, LA; ^2^ Department of Emergency Medicine, Ochsner Clinic Foundation, New Orleans, LA; ^3^ The University of Queensland Faculty of Medicine, Ochsner Clinical School, New Orleans, LA

**Keywords:** *Obstetric labor complications*, *patient simulation*, *simulation training*

## Abstract

**Background:** Simulation training improves the response to obstetric emergencies.

**Methods:** We review the current literature regarding simulation training for provider education, team training, and obstetric outcomes and describe the implementation of a multidisciplinary obstetric simulation program.

**Results:** A review of literature available at PubMed reveals many studies focused on provider education but few studies detailing the direct impact on patients. We review simulation reports that demonstrate improved clinical outcomes after obstetric emergencies—such as shoulder dystocia, postpartum hemorrhage, delivery of the second twin, operative vaginal delivery, urgent cesarean delivery, and neonatal resuscitation—as these studies formed the basis of the Ochsner Obstetrics and Gynecology Simulation Program in New Orleans, LA. We discuss the 3 principal simulation formats at Ochsner: a half-day course at the simulation training center, in-situ simulation on clinical care floors, and just-in-time training in the classroom. We also present detailed examples of simulation scenarios to assist others in creating a robust simulation program to ensure staff and providers are well trained to respond to obstetric emergencies.

**Conclusion:** The Ochsner Obstetrics and Gynecology Simulation Program was formulated on published literature and incorporates a variety of clinical settings, scenarios, and approaches to improve educational opportunities and response to obstetric emergencies.

## INTRODUCTION

Simulators have long been used as teaching tools in obstetrics and gynecology. Simulated birth using the Budin-Pinard phantom pelvic model was championed in 1898 during a presentation at the annual meeting of the Association of American Medical Colleges (AAMC) to provide resident training opportunities at a time when the majority of deliveries occurred in the home.^1^ In the 21st century, we are again experiencing a deficit in hospital-based learning opportunities, particularly in hands-on learning across all surgical specialties, and use of simulation-based training is expanding.^2^ The combination of simulated clinical scenarios and didactic learning has been shown to be more effective than didactics alone to maintain the knowledge base and acquire new skills such as teamwork and collaboration.^3^ In recognition of the value of simulation training, the Ochsner Clinical Simulation and Patient Safety Center opened in October 2017 with a mission to improve patient safety and clinical outcomes through delivery of high-quality experiential integrated team-based learning. In this article, we review the literature supporting obstetrics and gynecology simulation and describe the obstetrics and gynecology simulation training opportunities at Ochsner Health in New Orleans, LA.

## SIMULATION TRAINING

The military, the aviation industry, and the subspecialties of anesthesia and emergency medicine pioneered the use of simulation training and demonstration of competency, and in 2003, a Current Commentary in the journal *Obstetrics and Gynecology* encouraged obstetrics and gynecology programs to include training in the simulation laboratory.^4^ Publications describing obstetric simulation quickly followed, and a 2018 review reported that a search using the terms “obstetrics” and “simulation” identified 1,113 English-language articles in the PubMed database.^5^ An AAMC survey in 2011 revealed that obstetric simulation was being used by 93% of responding medical schools and 89% of responding teaching hospitals.^6^

Obstetric emergencies are high-acuity, low-frequency events, and effective response requires practice by both learners and experienced providers to achieve error-free execution. The American College of Obstetricians and Gynecologists supports simulation as an effective training modality to address educational needs and promote practice standardization.^7^

The Joint Commission also recognizes the importance of incorporating clinical drills to reduce the risk of neonatal injury and death. Beginning January 1, 2021, new standards for perinatal safety will require all accredited obstetric units to “conduct drills at least annually to determine system issues as part of ongoing quality improvement efforts.”^8^ Postpartum hemorrhage and hypertensive emergencies, the most common preventable causes of maternal morbidity and mortality, must be addressed in simulation training formats.^9^

Studies of several obstetric conditions amenable to simulation training have demonstrated objective evidence of the impact on patient outcomes. This literature formed the basis of the Ochsner simulation curriculum and is reviewed in the following sections.

### Shoulder Dystocia

Shoulder dystocia is an unpredictable critical emergency that requires immediate response to avoid fetal injury or death. In a birth complicated by shoulder dystocia, the fetal head delivers, but the anterior shoulder impacts behind the maternal symphysis pubis, delaying delivery and potentially resulting in neurologic injury, brachial plexus injury, and/or death. The obstetric provider requires immediate assistance to apply defined maneuvers to relieve the impaction, begin neonatal resuscitation, prepare for potential laparotomy, and inform the patient and her family of the critical nature of the clinical situation. Draycott et al reported an observational retrospective study of 39,000 births comparing management and neonatal outcomes complicated by shoulder dystocia before and after simulated training.^10^ Training encouraged prompt recognition and calls for help, the use of the McRoberts maneuver, application of suprapubic pressure, delivery of the posterior arm, or rotation of the fetal shoulders. Draycott et al found that after mandatory shoulder dystocia simulation for all personnel, the frequency of use of evidence-based maneuvers to relieve shoulder dystocia was higher, and the rate of neonatal brachial injury at birth was 4-fold lower (9.3% to 2.3%, relative risk 0.25, 95% confidence interval [CI] 0.11-0.57). Crofts et al found that improved management of shoulder dystocia after simulation training persisted for >12 months.^11^ Goffman et al also evaluated obstetrics and gynecology residents’ management of shoulder dystocia.^12^ Residents were scored on their use of initial (McRoberts maneuver, suprapubic pressure, gentle downward pressure) and advanced (Rubin maneuver, Wood corkscrew maneuver, delivery of the posterior arm) techniques and were given 1 point for each of the initial maneuvers and 1 additional point for appropriate use of a complex maneuver, for a maximum total score of 4. Residents demonstrated statistically significant improvement in mean maneuver scores (3.3 ± 0.9 vs 3.9 ± 0.4, *P*=0.001). Participants were also assessed for effective communication during the simulated shoulder dystocia. Communication tasks included recognizing and communicating shoulder dystocia to the team, calling for assistance, calling pediatrics, assuming a leadership role/instructing the team, and communicating with arriving help. Communication scores of residents and attending physicians improved significantly after training (residents, 3.5 ± 1.2 vs 4.9 ± 1.0, *P*<0.0001) (attending physicians, 3.6 ± 1.6 vs 4.9 ±1.1, *P*<0.0001).

### Postpartum Hemorrhage

Recognition of and response to postpartum hemorrhage also improve after simulation training. Marshall et al demonstrated significantly improved response times in the management of postpartum hemorrhage, including the recognition of postpartum hemorrhage, time to administer first uterotonic medication, performance of uterine massage, and time to administer second uterotonic medication.^13^ Medical management (appropriate use of 3 indicated medications) improved after training from 27.3% to 63.6% (*P*=0.01). In addition to the Joint Commission, the National Partnership for Maternal Safety: Consensus Bundle on Obstetric Hemorrhage strongly encourages drills and debriefing to improve patient outcomes in the event of postpartum hemorrhage.^14^

### Delivery of the Second Twin

In 2006 and in 2018, the American College of Obstetricians and Gynecologists issued and reaffirmed a committee opinion discouraging vaginal delivery of a term singleton breech but continuing to support vaginal delivery of a breech second twin, immediately decreasing vaginal breech delivery and training opportunities.^15^ Easter et al used simulation training for vaginal breech delivery and demonstrated improved trainee knowledge about and comfort with twin vaginal birth.^16^ They reported that knowledge about twin delivery improved (33.3% vs 58.3% questions correct, *P*<0.01), and personal comfort with performing breech extraction of a nonvertex second twin improved after the simulation (5.5% to 66.7%, *P*<0.01).

### Operative Vaginal Delivery

The rate of operative vaginal delivery has sharply decreased in the United States, from 9% of all deliveries in 1992 to 3.3% of all deliveries in 2013, resulting in far fewer training opportunities.^17^ Simulated forceps training has been shown to improve provider skill, knowledge, comfort, and patient outcomes.^18^ Following simulation training, Gossett et al demonstrated a lower incidence of third- and fourth-degree perineal lacerations after forceps-assisted vaginal delivery by residents (odds ratio [OR] 0.74, 95% CI 0.62-0.90), and Cheong et al demonstrated a decrease in cervical, severe labial, and high vaginal injury (OR 0.29, 95% CI 0.08-0.96); neonatal scalp injury (OR 0.14, 95% CI 0.02-0.98); and neonatal facial injury (OR 0.02, 95% CI 0.01-0.04) after simulation training.^19,20^

### Other Obstetric Emergencies

Cord prolapse—an obstetric emergency in which the umbilical cord emerges into the vagina before the vertex, resulting in significant risk of cord compression and fetal hypoxia—requires urgent cesarean delivery. Management of cord prolapse is improved after simulation-based training, with a statistically significant reduction in the interval of diagnosis to delivery from 25 to 14.5 minutes (*P*<0.001) and an increase in the proportion of cesarean deliveries during which recommended actions were performed (recognize cord prolapse, call for assistance, perform maneuvers to reduce pressure on the cord, communicate effectively with the patient, and take detailed contemporaneous documentation) from 34.78% to 82.35% (*P*=0.003).^21^ Severe preeclampsia, eclampsia, magnesium toxicity, vaginal delivery of a singleton breech with forceps, and repair of fourth-degree lacerations are all amenable to simulation, and management of these conditions significantly improves after simulation training.^22,23^

### Neonatal Outcomes

Draycott et al demonstrated the impact of shoulder dystocia simulation and teamwork training on neonatal outcomes, with improved 5-minute Apgar scores and reduced hypoxic-ischemic encephalopathy.^24^ Following training, infants born with 5‐minute Apgar scores ≤6 decreased from 86.6 to 44.6 per 10,000 births (*P*<0.001), and neonates with hypoxic-ischemic encephalopathy decreased from 27.3 to 13.6 per 10,000 births (*P*=0.032).

## OCHSNER OBSTETRICS AND GYNECOLOGY SIMULATION PROGRAM

The Ochsner Baptist Department of Obstetrics and Gynecology is an academic program providing level IV maternal/neonatal care and handling 3,750 births per year. The Ochsner Obstetrics and Gynecology Simulation Program is coordinated by a multidisciplinary group of obstetricians; anesthesiologists; neonatologists; obstetric nurse educators; nurse practitioners; and neonatal, obstetric, and critical care nurses. The Ochsner Obstetrics and Gynecology Simulation Committee conducts simulation exercises every other month and meets to review recent simulation efforts, to evaluate the anonymous evaluations and participant feedback solicited after each simulation, and to address any latent systemic safety issues identified during the group debrief. Members of the committee discuss topics and scheduling of simulation events, including educational goals, scenarios, and appropriate timing and location. The committee is careful to include learners with different levels of expertise from all shifts and clinical locations in the hospital.

The Ochsner Obstetrics and Gynecology Simulation Committee includes several members who are formally trained in simulation, with experience incorporating simulation into learning opportunities. Scenarios are tailored to the learning objectives and location. Careful attention is paid to creating a safe space for simulation learning. Jenny Rudolph's process of “debriefing with good judgment,” whereby adequate time is planned to review the learning points of the exercise and participants actively engage in discussion of “what went well and what we should change,” is a cornerstone of the program.^25^

### High-Fidelity Obstetrics and Gynecology Simulation

**Figure 1.**
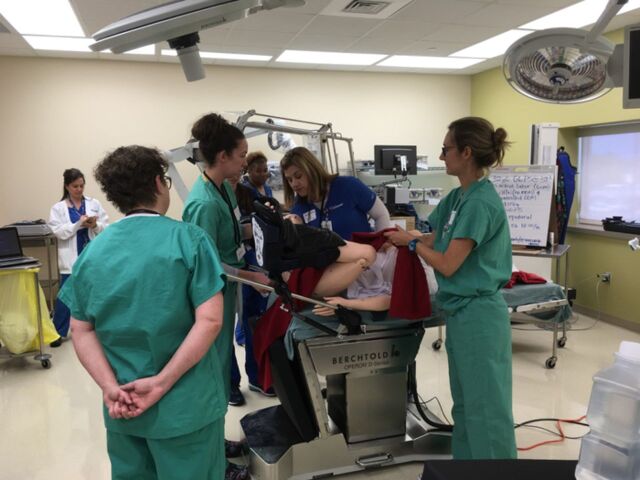
**The Ochsner Clinical Simulation and Patient Safety Center is equipped with high-fidelity models capable of simulated birth and advanced maternal and neonatal resuscitation. Here, trainees participate in a postpartum hemorrhage simulation.**

**Figure 2.**
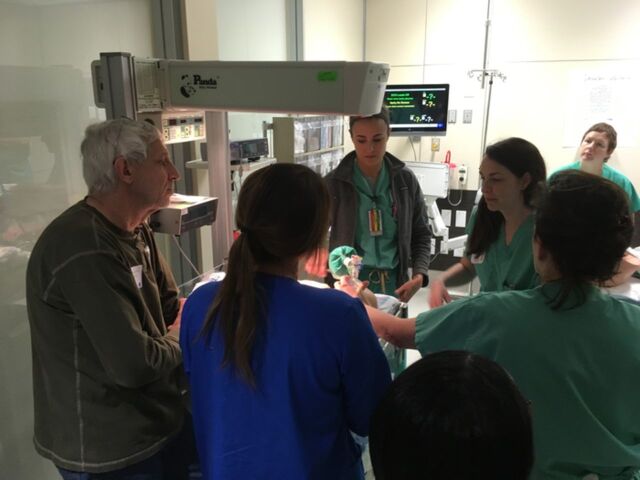
**The mission of the Ochsner Clinical Simulation and Patient Safety Center is to improve patient safety and clinical outcomes through delivery of high-quality experiential integrated team-based learning. Here, participants learn critical lifesaving skills during a simulated neonatal resuscitation.**

The Ochsner Clinical Simulation and Patient Safety Center, located at the main campus of Ochsner Health in New Orleans, LA, is equipped with high-fidelity models capable of simulated birth and advanced maternal and neonatal resuscitation. An annual voluntary multidisciplinary simulation course incorporates didactics and hands-on response to obstetric emergencies. Participants include obstetricians, anesthesiologists, neonatologists, emergency medicine physicians, midwives, and obstetric and anesthesiology residents, with 24 participants per course divided into 8-person groups. Didactics are presented as an inverted classroom and completed prior to the hands-on course. Topics covered include maternal cardiac arrest with resuscitative cesarean delivery, postpartum hemorrhage with massive transfusion (Figure 1), and shoulder dystocia, including neonatal resuscitation (Figure 2), management of vaginal breech, and code stroke. Each half-day course includes 3 simulations that are run twice during a 45-minute period, as well as prebriefs, debriefs, and time for participant feedback.

### In-Situ Simulation

Supplementing the training offered at the simulation center, the Ochsner Obstetrics and Gynecology Simulation Committee coordinates in-situ simulation that “occurs in the actual clinical environment, [and] whose participants are on-duty clinical providers during their actual workday.”^26^ In addition to improving knowledge, skills, and team-based communication for managing obstetric emergencies, in-situ simulation allows nurses, residents, midwives, and staff attendings to manage simulated emergencies in their usual clinical environment.^27^ While in-situ simulations provide a rich opportunity for clinical training, the acuity of the labor and delivery unit and the unscheduled nature of in-situ simulation can pose a challenge. The committee has determined that long, complex scenarios such as maternal cardiac arrest with resuscitative cesarean delivery are best presented at the simulation center. Short, targeted scenarios, such as medical management of postpartum hemorrhage are planned for in-situ training.

In-situ simulation has proven effective at identifying latent systems issues that hamper effective emergency response. For example, simulation of a severe hypertension in a recently opened antepartum unit revealed that the bedside code buttons had not been activated. A simulated umbilical cord prolapse in the alternative birth center revealed that providers did not carry the key required to gain entrance. An eclampsia simulation disclosed that the magnesium dose stocked in the medication dispensing system was inadequate for treatment following a seizure. In addition to planning and executing simulation exercises, the Ochsner Obstetrics and Gynecology Simulation Committee advocates for solutions to any barriers identified, which has improved enthusiasm for simulation among department members. In-situ simulation topics, locations, and examples of barriers identified are listed in the Table.

**Table.** Examples of Ochsner Baptist In-Situ Simulations

Topic	Location	Safety Barrier Identified
Placental abruption	Labor and delivery unit	Communication via personal cell phones found ineffective for emergencies
Eclamptic seizure	Antepartum	Attendings not receiving code notification
Eclamptic seizure	Obstetric emergency room	Magnesium not stocked in Pyxis
Eclamptic seizure	Postpartum	Wrong number for code operator; lorazepam not stocked in Pyxis
Severe hypertension	Antepartum	Code button deactivated
Precipitous preterm delivery	Antepartum	Unknown location of warmer; unable to override elevator
Precipitous delivery	Obstetric emergency room	Stat pack inadequate
Postpartum hemorrhage	Operating room	Massive transfusion protocol order set via telephone communication improved response time
Cord prolapse	Alternative birth center	Requires key to access; bed does not fit through front door
Car delivery	Garage	“Code stork” confused with “code stroke”
Malignant hyperthermia	Operating room	Resuscitation box disorganized
Fire	Operating room	Location of oxygen shutoff unknown
Malignant hyperthermia	Operating room	Optimal medication not available at Ochsner Baptist

### Just-in-Time Simulation

Just-in-time training—simulation provided directly before an actual clinical procedure—is an effective tool for residency training.^28^ Just-in-time topics at Ochsner Baptist include shoulder dystocia, operative vaginal delivery, vaginal breech delivery, application of Piper forceps, delivery of the second twin, and postplacental insertion of an intrauterine device.

### Classroom Simulation

Incorporation of simulation exercises into didactic lectures can increase the interest level of both the learners and the lecturer. A study of fourth-year medical students during a critical care rotation found that compared to the traditional lecture format, students ranked simulation-based teaching higher with regard to enjoyment (*P*=0.0044), interest (*P*=0.0068), relevance to taught subject (*P*=0.0313), ease of understanding (*P*=0.0476), and accessibility to posing questions (*P*=0.001).^29^ At Ochsner Baptist, residents, medical students, nurses, and paramedics participate in lectures covering topics such as team training and communication, obstetric emergencies, and precipitous delivery. The lectures incorporate simulation opportunities in the classroom, using role play and pelvic models.

The Ochsner Obstetrics and Gynecology Simulation Program uses both low- and high-tech equipment for a variety of simulation exercises. High-fidelity birthing mannequins allow for realistic representation of complex emergencies (such as cardiac arrest after amniotic fluid embolus) and facilitate training for an effective multidisciplinary response. However, most in-situ simulations incorporate live patient actors who use birthing props such as the MamaNatalie birthing simulator (Laerdal Medical). Such props were originally designed for teaching in low-resource settings; the patient actor wears the device on the chest and stomach and can interact in a realistic way with students while simulating precipitous birth, postpartum hemorrhage, and other emergencies. Use of the live actor emphasizes the importance of effective communication skills between the patient and learner.

## SIMULATION BARRIERS

A survey of staff anesthesiologists at an academic center in Canada identified time and financial issues as the biggest barriers to the use of simulation-based education.^30^ Ideally, simulation occurs in small groups, with adequate time for multiple sessions to ensure effective learning opportunities for all department members.

The annual course offered at the Ochsner Clinical Simulation and Patient Safety Center requires a half-day away from work, and learners are compensated for their time at the center, posing a financial barrier. Continuing medical education and continuing education unit credits are awarded to participants.

In-situ simulation occurs during working hours while providers are responsible for actual patient care needs, so time for simulation is in short supply. In-situ simulation activity must sometimes be postponed because of unit acuity, particularly with limited staff on night shifts. The cost of in-situ simulation is minimal, as we use recycled or expired equipment.

An additional barrier to expansion of simulation opportunities is the lack of objective outcomes. Data validating the impact of the simulation training at Ochsner are challenging to collect, as we focus on rare but life-threatening events. A future goal is to collect data regarding emergency response before and after simulation training.

Much of the success and acceptance enjoyed by the simulation program at Ochsner can be attributed to the committee's focus on patient safety and education and the commitment to address potential latent systemic deficiencies that the exercises bring to light. A second-year obstetrics and gynecology resident's first encounter with shoulder dystocia is an example of the program's success. The resident used a complex maneuver to deliver the posterior arm and delivered the baby unharmed. She attributed her success to the just-in-time simulation she had practiced that morning, and she is now an enthusiastic supporter of simulation.

## CONCLUSION

Simulation-based learning using pelvic models has been part of obstetric training for more than a century. The literature provides robust support for improved patient outcomes after simulation-based learning for management of obstetric emergencies. The multidisciplinary simulation program at Ochsner Health is based on this literature and works toward improving team communication, resident education, and patient safety by using a variety of simulation settings, techniques, and scenarios. Future efforts will incorporate data collection to show the impact of simulation training on patient outcomes.

## Notes

This article meets the Accreditation Council for Graduate Medical Education and the American Board of Medical Specialties Maintenance of Certification competencies for Patient Care and Medical Knowledge.
